# Epigenetic Features in Newborns Associated with Preadolescence Lung Function and Asthma Acquisition during Adolescence

**DOI:** 10.3390/epigenomes8020012

**Published:** 2024-03-22

**Authors:** Mohammad Nahian Ferdous Abrar, Yu Jiang, Hongmei Zhang, Liang Li, Hasan Arshad

**Affiliations:** 1 Division of Epidemiology, Biostatistics, and Environmental Health, School of Public Health, University of Memphis, Memphis, TN 38152, USA; mabrar@memphis.edu (M.N.F.A.); yjiang4@memphis.edu (Y.J.); liang.li@shelbycountytn.gov (L.L.); 2 David Hide Asthma and Allergy Research Centre, Isle of Wight P030 5TG, UK; s.h.arshad@southampton.ac.uk; 3 Human Development and Health, Faculty of Medicine, University of Southampton, Southampton S017 1BJ, UK

**Keywords:** DNA methylation, lung function, asthma acquisition, FEV1, FVC, FEV1/FVC ratio, IOWBC, ALSPAC

## Abstract

The association between newborn DNA methylation (DNAm) and asthma acquisition (AA) during adolescence has been suggested. Lung function (LF) has been shown to be associated with asthma risk and its severity. However, the role of LF in the associations between DNAm and AA is unclear, and it is also unknown whether the association between DNAm and AA is consistent with that between DNAm and LF. We address this question through assessing newborn epigenetic features of preadolescence LF and of AA during adolescence, along with their biological pathways and processes. Our study’s primary medical significance lies in advancing the understanding of asthma’s early life origins. By investigating epigenetic markers in newborns and their association with lung function in preadolescence, we aim to uncover potential early biomarkers of asthma risk. This could facilitate earlier detection and intervention strategies. Additionally, exploring biological pathways linking early lung function to later asthma development can offer insights into the disease’s pathogenesis, potentially leading to novel therapeutic targets. Methods: The study was based on the Isle of Wight Birth cohort (IOWBC). Female subjects with DNAm data at birth and with no asthma at age 10 years were included (n = 249). The R package ttScreening was applied to identify CpGs potentially associated with AA from 10 to 18 years and with LF at age 10 (FEV1, FVC, and FEV1/FVC), respectively. Agreement in identified CpGs between AA and LF was examined, along with their biological pathways and processes via the R function gometh. We tested the findings in an independent cohort, the Avon Longitudinal Study of Parents and Children (ALSPAC), to examine overall replicability. Results: In IOWBC, 292 CpGs were detected with DNAm associated with AA and 1517 unique CpGs for LF (514 for FEV1, 436 for FVC, 408 for FEV1/FVC), with one overlapping CpG, cg23642632 (*NCKAP1*) between AA and LF. Among the IOWBC-identified CpGs, we further tested in ALSPAC and observed the highest agreement between the two cohorts in FVC with respect to the direction of association and statistical significance. Epigenetic enrichment analyses indicated non-specific connections in the biological pathways and processes between AA and LF. Conclusions: The present study suggests that FEV1, FVC, and FEV1/FVC (as objective measures of LF) and AA (incidence of asthma) are likely to have their own specific epigenetic features and biological pathways at birth. More replications are desirable to fully understand the complexity between DNAm, lung function, and asthma acquisition.

## 1. Introduction

Asthma is the second most common chronic respiratory disease, affecting individuals across all ages worldwide. It is estimated that around 300 million people grapple with this condition, and the disease burden is projected to increase further [1,2]. Asthma acquisition denotes the process by which an individual, who previously did not experience the condition, develops or acquires asthma. The stage of adolescence signifies a pivotal transition from childhood to adulthood. Numerous risk factors have been identified in association with asthma acquisition, such as genetic, epigenetic, environmental, and lifestyle factors, etc. [3,4].

Epigenetic mechanisms such as DNA methylation (DNAm), the addition of methyl groups to cytosine where cytosine-phosphate-guanine (CpG) dinucleotides occur in the DNA sequence, regulates both gene expression and splicing resulting in production of different messenger RNAs (mRNA) [5]. DNAm has been shown to be associated with childhood asthma [6,7]. Furthermore, our previous findings suggested a potential association between DNAm at birth and the acquisition of asthma during adolescence [8].

Lung development begins in early gestation and continues to complete maturation at about the age of eight years. Lung growth is thought to finish in late adolescence or early adulthood when lung function reaches its maximum level and gradually diminishes. Lung function refers to the capacity of the lungs to move air in and out efficiently, enabling the exchange of oxygen and carbon dioxide between the bloodstream and the external environment. It is a crucial measure of respiratory health and is commonly assessed through various lung function tests. During adolescence, lung function undergoes significant changes, such as the lungs and chest wall continuing to grow and the respiratory muscles becoming stronger. The lung function is an important predictor in assessing the development of asthma or related diseases. Pulmonary function tests, commonly referred to as lung function tests, are tests assessing how well the lungs are performing. Lung function tests are frequently used to diagnose and assess the severity of diseases and health disorders such as lung cancer [9], chronic obstructive pulmonary disease [10], asthma [11], and other respiratory infections. Different tests like spirometry, long volume, gas diffusion, among others, are used to evaluate lung functions. Most commonly used lung function parameters are spirometry measurements of forced vital capacity (FVC), forced expiratory volume in one second (FEV_1_), and their ratio (FEV_1_/FVC) representing different physiological and clinical conditions [12]. 

Numerous factors may affect the association between asthma acquisition and lung function. There is growing epidemiological evidence that environmental influences in the early years of life significantly impact lung health and maximal lung function. These potential determinants include individual factors, genetic and epigenetic factors, early life events, allergic diseases, environment, and lifestyle. Smoking has been identified as a factor that increases the risk of asthma and decreases lung function [13]. An Australian birth cohort found that parental smoking in utero increased the risk of asthma and deteriorated lung development in their offspring by adolescence [14].

Studies on DNAm at an earlier age and lung function levels or asthma status in later life are relatively limited. By examining individual CpG sites and differentially methylated regions at birth, eight CpG sites were identified as potential epigenetic biomarkers for asthma acquisition during adolescence [15]. Another study that investigated the association between DNA methylation at birth and asthma acquisition showed that the associated CpGs were more likely to be different between genders and may also be unique to specific transition periods [8]. Additionally, a separate study highlighted the role of atopy as a mediator in the relationship between DNA methylation and asthma development during both pre- and post-adolescence [16].

Only limited research investigated the association between DNAm at birth and lung function in adolescence. Puberty, physiological development, and frequent increase in BMI are some of the vital gender-dependent changes that occur during the preadolescence and adolescence stages. Additionally, it is a crucial period for the development of lung function [17]. One of our recent study has shown an association of DNA methylation at birth with lung function development until age 26 years [18]. This study has identified CpGs significantly associated with lung function at age levels 10 and 18 and has found 8 CpGs on genes involved in the developmental process of lung functions [18]. Another a more in-depth study investigated the association between DNA methylation and the three different types of lung functions for each gender. This study showed that, CpG sites were associated with a change in the modified Tiffeneau-Pinelli index lung function (FEV_1_/FVC ratio) but not with FEV_1_ or FVC for females [19]. DNA methylation has also been shown to be linked with the lung function of non-smokers [20,21].

The primary objective of this paper is to investigate whether epigenetic features observed in newborns are associated with preadolescence lung function and examine whether these identified epigenetic features are linked to the risk of asthma acquisition during adolescence in the same cohort. Furthermore, we aim to explore the potential biological pathways that may underlie the relationship between early lung function and adolescent asthma development. This research seeks to shed light on the epigenetic mechanisms that may underlie the interplay between early lung function and asthma development, contributing to a deeper understanding of asthma pathogenesis. We have validated our findings using an independent dataset to strengthen the reliability of our results.

## 2. Results

Within IOWBC, 405 girls were asthma-free at age 10 (complete samples), of whom 249 had DNAm data at age 10 years (study samples). Of these 249 girls, at age 18 years, 19 (7.63%) developed asthma. We compared with the complete samples on the variables of interest including asthma acquisition, lung function parameters, and height. None of these variables in the study samples exhibited statistically significant differences from the complete samples (Table 1). Data from the 249 subjects were included in subsequent analyses.

Our assessment of the association between lung function at age 10 and asthma acquisition during adolescence indicated a statistically significant association with FEV1 (*p*-value = 0.0181) but not with FVC (*p*-value = 0.345) or FEV1/FVC (*p*-value = 0.0634).

We further compared the difference in DNAm at birth at each of the quality controlled 551,710 CpG sites between subjects who developed asthma at age 18 years and those who were asthma-free, using the ttScreening R package. In total, DNAm at 292 CpG sites were shown to be associated with asthma acquisition. To gain deeper insights into the role of lung function in the association of DNAm and asthma acquisition, we conducted a mediation analysis to investigate whether lung function at age 10, measured by FEV1, FVC, and the FEV1/FVC ratio, serves as a mediator in the relationship between DNA methylation at birth and the acquisition of asthma between ages 10 and 18. Among the 292 identified CpGs showing association with asthma acquisition, none of the lung function parameters played a role as a potential mediator (all *p*-values > 0.05).

**Figure 1:**
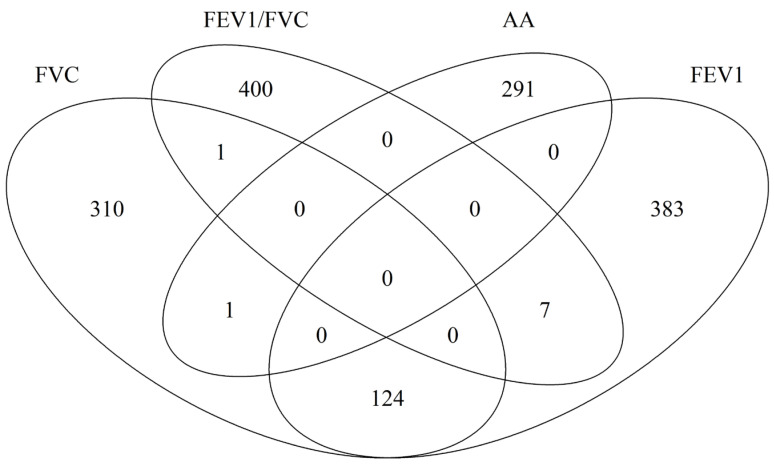
Overlap of identified CpGs which DNA methylation at birth are associated with asthma acquisition (AA), and lung function measures including FEV1, FVC, and FEV1/FVC ratio.

**Figure 2:**
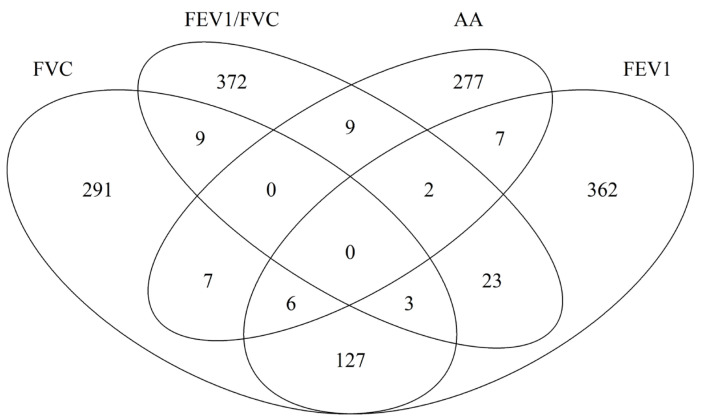
Overlap of genes that have CpGs with DNA methylation at birth associated with asthma acquisition, and lung function measures including FEV1, FVC, and FEV1/FVC ratio.

We then independently examined each of the 551,710 CpG on its association in DNAm with lung function at age 10 years. In total, 436 CpG sites were identified for FVC, 514 for FEV1, and 408 for FEV1/FVC. The number of CpGs overlapped between FVC and FEV1 124 common CpGs (Figure 1 and Supplementary File S1). No CpGs were common across all the three types of lung function measurements. For the CpGs identified for the three lung function parameters, only one showed statistically significant association with asthma acquisition, cg23642632. This CpG is on the gene of NCKAP1. Since one CpG site can possibly regulate the functionality of more than one gene, for the Identified CpGs, we further assessed their mapped genes common between different lung function parameters and between lung function and asthma acquisition (Figure 2). The largest overlap is still between FVC and FEV1 (136 common genes) and 39 genes common between at least one of the three lung function parameters and asthma (Supplementary File S1).

We further examined the features of biological pathways for the genes to which the identified CpGs were mapped. This analysis was conducted for each of the three lung function parameters and for asthma acquisition. The top 10 pathways which have the smallest *p*-value for each outcome are shown in Table 2. The pathways that have raw *p*-values smaller than 0.05 can be found in Supplementary File S3. For asthma transition, the top 10 pathways are more related to postsynaptic and trans-synaptic signaling. For FVC, among the top 10 pathways, three of them related to hormone secretion, which are “positive regulation of peptide hormone secretion”, “positive regulation of peptide secretion”, “positive regulation of insulin secretion”, and “positive regulation of hormone secretion“. Some hormones, such as cortisol and epinephrine, influence airway tone and inflammation, consequently affecting lung function [22,23]. For FEV1, the most related pathway is mannosyltransferase complex, which adds mannose to substrate proteins at the endoplasmic reticulum. For ratio, the most associated pathway is about ketone biosynthetic process. Among the top 10 pathways for asthma transition and those for each of the lung function parameters, we observed no overlap between asthma transition and any of the lung function measures.

**Figure 3:**
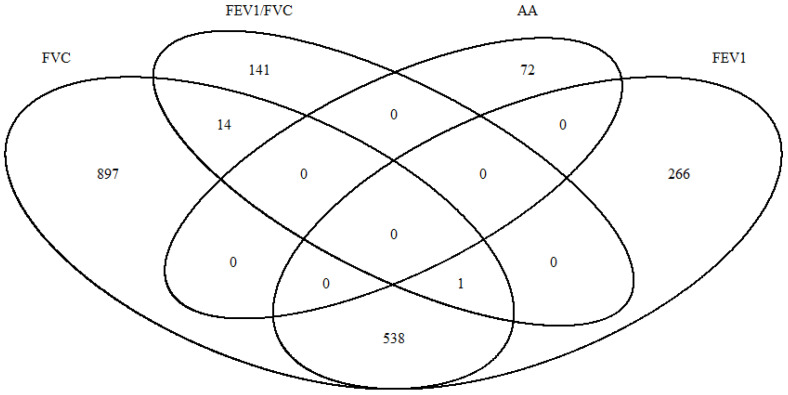
Overlap of identified CpGs which DNA methylation at birth are associated with asthma acquisition (AA), and lung function measures including FEV1, FVC, and FEV1/FVC ratio in ALSPAC cohort.

We further tested our findings in an independent cohort, the ALSPAC birth cohort, which included 196 girls who were asthma-free at age 7.5, with 17 developing asthmas by the age of 16.5 years. In ALSPAC, there are, in total, 48,2855 CpG sites with DNA methylation measured in cord blood. Among them, 30,1854 CpGs are also available in IOWBC. We conducted an independent ttScreening analysis from ALSPAC cohort for the 30,1854 Cpgs for each of the lung functions and asthma acquisition. In total, 72 CpG sites were identified as significantly associated with asthma transition. None of these CpGs are found to be associated with lung function measures. In ALPAC cohort, we identified 804 CpGs associated with FEV1, 1450 with FVC, and 156 with FEV1/FVC (Figure 3). Among lung function, FEV1 and FVC has the largest overlapped CpGs, which is 539.

Among the 292 CpGs identified in IOWBC with DNAm associated with asthma acquisition, 174 CpGs were available in the ALSPAC cohort, of which DNAm at 76 (43.68%) CpGs shared the same directions of association as those in IOWBC (Table 3). The regression coefficients and *p*-values for the replication study are available in Supplementary File S2. For lung function FEV1/FVC ratio, 134 of the 226 (59.29%) IOWBC-identified CpGs showed the same direction of associations in ALSPAC (Table 3). Among the 11 CpG that are significant both in IOWBA and ALSPAC cohort, 72.73% show the same direction. For the IOWBC-identified CpGs, FVC shows the largest number of CpGs (24 out of 222) that are also significantly associated with DNA methylation at birth. As shown in Figure S1 (Supplementary File S4), the replicated CpGs do not overlap between asthma acquisition and lung function measures. Additionally, we identified the top 10 pathways for asthma acquisition and each lung function in the replication cohort (Table S1 in Supplementary File S4).

## 3. Discussion

The present study explores potential associations between epigenetic characteristics observed in newborns and lung function in preadolescence, and whether these identified epigenetic traits are connected to the risk of asthma acquisition during adolescence. We observed a statistically significant association between lung function FEV1 at age 10 and asthma acquisition status. There are a large number of overlapped CpGs between FEV1 and FVC. This result is not surprising, given the established highly positive correlation between FEV1 and FVC [24]. However, this association was not observed for lung function measures FVC and the FEV1/FVC ratio. Additionally, our mediation analysis showed that lung function at age 10 does not act as a mediator between DNAm in newborns and the development of asthma during adolescence. The absence of overlapping CpG sites for DNAm related to lung function at age 10 and those associated with asthma acquisition suggests that these processes involve distinct biological pathways or mechanisms. Thus, asthma acquisition during adolescence is probably driven by factors during adolescence such as puberty/hormone, height gain, medication, or smoking, rather than lung growth during childhood.

Asthma is a chronic respiratory condition characterized by airway inflammation, bronchoconstriction, increased mucus production, and heightened airway reactivity. Asthma acquisition is a complex process that involves airway constriction and inflammation, immune responses, and allergic reactions. Various inflammatory pathways play an important role in the development of asthma [25]. On the other hand, lung function refers to the overall capacity of the lungs to move air in and out for the exchange of oxygen and carbon dioxide. It includes the ability to inhale and exhale, airway resistance, and gas exchange. Many biological processes affect lung function, such as airway smooth muscle contraction, airway epithelial function, gas exchange pathways, metabolic pathways, hormonal and endocrine pathways, and inflammatory pathways [26,27]. In the current study, the lack of agreement in epigenetics and related pathways between pre-adolescence lung function and asthma acquisition reflects that lung development and asthma acquisition may be through different pathways linked to respiratory health, and subsequent studies are needed to further examine this observation.

We recognize that the prenatal exposures, such as folate supplementation and maternal smoking, are important factors that influence the risk of asthma and impact lung function. Epigenetics has been suggested as a potential marker of past exposures or notable changes in life. Given that our study measures DNAm at birth, following the chronological order, we expect that the influence of prenatal exposures has been reflected in DNAm at birth. Under this consideration, we did not include prenatal exposures in the analysis to avoid double dipping. In addition, postnatal risk factors for adolescent asthma, such as obesity, were also excluded. This analysis strategy aligns with the objective of the study; that is, establishing a connection between DNAm at birth and asthma acquisition (between ages 10 to 18 years) or lung function (at age 10) during the period of adolescence transition. Under this study design, postnatal risk factors such as obesity are more likely to serve as an intervening variable rather than a confounder between DNAm at birth and the two outcome variables (asthma acquisition and lung function).

This study exclusively focuses on female participants. The absence of studies on male subjects in the current analysis is due to the small number of cases in asthma acquisition of males during adolescence in IOWBC and ALSPAC cohort [15]. Among the subjects with data available for analysis, only several males had asthma acquisition (and thus rare events). Existing ttScreening methods do not offer reliable inferences with rare events. To streamline our analysis and minimize complexity, the current study focuses on females. Our previous study showed that lung function development is different between male and female. Lung development occurs during and until the end of puberty for males whereas for female, lung development is almost completed following menarche [28]. Future studies are desired to incorporate both males and females into the assessment given the gender difference in lung faction development and asthma at all ages. Doing so will also enable an exploration of possible shared patterns of biological pathways and associations between the genders. Nevertheless, our findings indicate potentially distinct epigenetic signatures between lung function development and asthma acquisition, and thus support the idea that lung function is likely not a causal factor for asthma acquisition.

## 4. Materials and Methods

### 4.1. Study Population

This study utilize data from the Isle of Wight Birth Cohort (IOWBC). The initial cohort was comprised of individuals born between January 1989 and February 1990 [29]. These participants have been followed up since birth until 26 years old. For the present study, we focused on females who were free of asthma at the age of 10, and their asthma status was assessed at the age of 18. The total number of subjects is 405. Among these 405 girls, 249 had DNA methylation measured at birth. Therefore, the sample size of our study was 249.

### 4.2. Asthma Acquisition

Asthma status was evaluated using the International Study of Asthma and Allergies in Childhood (ISAAC) questionnaires at ages 10 and 18. Asthma acquisition during adolescence was defined as asthma-free at age 10 and acquiring asthma at age 18.

### 4.3. Height Adjusted Lung Function

We have three types of lung functions (FEV1, FVC, and ratio of FEV1/FVC) at age 10 for this study. FEV1 (forced expiratory volume) is defined as the volume of air that a person can exhale during a forced breath in 1 s. FVC (Forced Expiratory Volume) is defined as the total amount of air exhaled by a subject during the FEV test. Previous studies have shown an association between height growth in puberty and lung functions [30,31]. To adjust the confounding effect of height, we regressed lung function measurements FEV1 and FVC on height at age 10. The residual values for FEV1 and FVC were used for all the subsequent studies.

### 4.4. DNA Methylation at Birth

DNA was isolated from dried blood spots on Guthrie cards, which is collected within seven days after from a heel prick of the kid, using a method based on the procedure described by Beyan et al. [32]. The standard salting out procedure was used to isolate DNA and its concentration was determined by Qubit quantitation. One microgram of DNA was bisulfite-treated for cytosine to thymine conversion using the EZ 96-DNA methylation kit (Zymo Research, Irvine, CA, USA), following the manufacturer’s standard protocol. The Infinium EPIC BeadChip (EPIC array) (Illumina, San Diego, CA, USA) was used to measure the levels of DNA methylation at the genome scale, using the manufacturer’s standard protocol. The R package ComBat [33] was used to remove batch effects in the combined dataset. The CpG sites with probe-SNPs within ten base pairs and with minor allele frequency (MAF) greater than 0.007 (representing ~10 subjects in the complete study cohort) were excluded, as were previously identified cross-reactive probes. Overall, DNA methylation of 551,710 autosomal CpGs were included for analysis. Beta-values were converted to M-values using base 2 logit-transformation.

To estimate cell composition for each sample, we used the function estimateCellCount in R-package minfi [34]. To adjust for effect of cell type, the methylation of CpGs (M-value) was regressed on the proportion of the cell types (CD4+ T cells, natural killer cells, neutrophil, B cells, monocytes, and eosinophils). The residuals not explained by cell types were estimated for each sample. These estimated residuals were used in the following analysis.

### 4.5. Statistical Analysis

**Descriptive statistics**

We summarized the demographic characteristics of the original population (n = 405) and the 249 subjects used for the current study for categorical variables with frequency and percentages and continuous variables with mean and standard deviation (SD), respectively. To compare the sample with the original cohort, one sample z-test was used to compare proportions, and one sample t-test was used to compare the mean.

Using the CpGs identified in IOWBC, we conducted logistic regression to evaluate the association between DNA methylation at birth with different outcomes, asthma acquisition, and lung function at age 8, including the FEV1, FVC, and FEV1/FVC ratio. For all the analysis, we have used RStudio 4.2.2 version.

**ttScreening**

In our study, we focused on the association between DNA methylation and the outcomes of interest (asthma acquisition and lung function). Instead of using any filter of effect size for DNA methylation, we use ttScreening to identify CpGs that are significantly associated with outcome. ttScreening is an R package that uses training and testing data to screen the CpGs sites whose DNA methylation has a significant association with the variable of interest on the whole genome [35]. Initially, the ttScreening approach was employed to identify CpG sites associated with asthma acquisition. For this analysis, CpG sites that had a passing rate of 50% or higher were considered to be significant. The passing rate here is defined as the minimum frequency required for a DNA methylation site (in our case, CpG sites) to be treated as an informative site. During the screening process, 100 training/testing analyses are done, and after each training/testing process, the status of each DNA methylation site being selected is recorded. This training/testing process is closely related to the concept of cross-validation, and a CpG site with a higher passing rate is likely to be more informative. We used 50% following the suggestion by Ray et al. [35].

The mapped genes of identified CpGs were located using the Illumina manifestation file, as well as the University of California Santa Cruz (UCSC) Genome Browser Database [36].

**Mediation analysis**

For CpGs that are significantly associated with asthma acquisition, we further conducted a mediation analysis. DNA methylation at birth was treated as an exposure variable. Each of the lung function measures was treated as a mediation variable, and asthma acquisition as the outcome. We followed standard steps in mediation analyses using the R lavaan package [37]. Both direct and indirect effects of DNAm at birth were evaluated on its association with asthma acquisition during adolescence, with lung function at age 10 as the mediator.

**Pathway analysis and mapping**

The selected CpGs are further evaluated for gene ontology to identify pathways and biological processes. This analysis was conducted using the Gene Ontology Testing for Illumina Methylation Array Data (GOMETH) approach, which is implemented through the R package missMethyl, gometh function [38]. The GOMETH analysis was carried out to identify pathways and biological processes associated with each set of CpGs that successfully passed the ttScreening. We have used the UCSC genome browser to locate respective genes associated with significant CpG sites found from our study. For the extraction of information, we have Ilumina ID and the location of chromosomes retrieved from GOMETH analysis.

### 4.6. Replication Study

Our study also uses a replication cohort (ALSPAC) to acquire the consistency of results obtained from the discovery cohort (IOWBC). The Avon Longitudinal Study of Parents and Children (ALSPAC) birth cohort is the replication cohort. ALSPAC is a prospective birth cohort investigating the effect on health and development across the life cycle through data collection of multiple generations [39]. The sample size for the replication study is 139. Cord blood was used to extract DNA for DNA methylation measurement. The lung functions are measured at the age of 8. Asthma acquisition during adolescence was defined as asthma-free at age 7.5 and acquiring asthma before 16.5 years old.

## 5. Conclusions

The current study implies that lung function (LF) and asthma (AA) acquisition during adolescence are likely to possess distinct epigenetic characteristics and biological pathways at birth. Further replications are needed to better understand the relationships of DNA methylation (DNAm) and lung function with the development of asthma.

## Figures and Tables

**Table 1:** Summary of demographics in IOWBC.

	IOWBC (n = 405)	Sample (n = 249)	*p*-Value
**Variables**	**Count (%)**	**Count (%)**	
Asthma acquisition	30 (7.41%)	19 (7.63%)	0.8943
	**Mean (SD)**	**Mean (SD)**	
FEV1 (L)	2.01(0.29)	2.01(0.31)	0.8340
FVC (L)	2.253 (0.33)	2.25(0.34)	0.9572
FEV1/FVC Ratio	0.89 (0.056)	0.90(0.054)	0.1668
Height (cm)	139.33 (6.51	139.39(6.55)	0.8947

FVC: forced vital capacity; FEV1: forced expiratory volume in 1; SD: standard deviation.

**Table 2:** Top 10 pathways in IOWBC.

FVC	FEV1	Ratio	Asthma Acquisition
positive regulation of peptide hormone secretion	mannosyltransferase complex	ketone biosynthetic process	postsynaptic density membrane
positive regulation of peptide secretion	dorsal/ventral axon guidance	corticotropin secretion	flavonoid glucuronidation
glycosylphosphatidylinositol-N-acetylglucosaminyltransferase (GPI-GnT) complex	MHC class II protein complex binding	basolateral plasma membrane	trans-synaptic signaling by lipid modulating synaptic transferase
positive regulation of insulin secretion	MHC protein complex binding	C21-steroid hormone biosynthetic process	trans-synaptic signaling by endocannabinoid
positive regulation of cytosolic calcium ion concentration involved in phospholipase C-activating G protein-coupled signaling pathway	intrinsic component of endosome membrane	Ctf18 RFC-like complex	xenobiotic glucuronidation
structural constituent of muscle	regulation of synaptic transmission, cholinergic	olefinic compound biosynthetic process	postsynaptic specialization membrane
positive regulation of hormone secretion	polymeric immunoglobulin receptor activity	response to osmotic stress	catalytic activity, acting on a protein
signaling receptor regulator activity	regulation of elastin biosynthetic process	cell–cell contact zone	ionotropic glutamate receptor binding
phospholipase C-activating G protein-coupled receptor signaling pathway	negative regulation of elastin biosynthetic process	basal plasma membrane	ubiquitin-like protein transferase activity
positive regulation of secretion by cell	negative regulation of synapse assembly	DNA clamp loader activity	integral component of postsynaptic density membrane

**Table 3:** Results from ALSPAC replication cohort.

	IOW (CpG Sites from Ttscreening)	No. of Common CpG Sites among IOW and ALSPAC	No. of Coefficients with Same Direction (All Common CpG Sites)	No of Significant CpG Sites from ALSPAC (Raw *p*-Value)	No of Coefficients with Same Direction (All Significant CpGs from ALSPAC)
FEV1	514	282	132 (46.81%)	21	7 (33.33%)
FVC	436	222	108 (48.65%)	24	12 (50%)
Ratio	408	226	134 (59.29%)	11	8 (72.73%)
Asthma acquisition	292	174	76 (43.68%)	8	0 (0%)

## Data Availability

The data used in the current study will be available upon request from the corresponding author with justification due to privacy and ethical restrictions. Data can also be requested through this site: https://allergyresearch.org.uk/studies/birth-cohort/#cohort-data-use (accessed on 19 March 2024).

## Supplementary Materials

The following supporting information can be downloaded at: https://www.mdpi.com/article/10.3390/epigenomes8020012/s1, File S1: selected CpGs; File S2: replicated CpGs; File S3: pathways; File S4: top 10 pathway ALSPAC.
