# Supplementary material for: Epigenetic Features in Newborns Associated with Preadolescence Lung Function and Asthma Acquisition during Adolescence
Source: Epigenomes. 2024 Mar 22;8(2):12. doi: 10.3390/epigenomes8020012 (PMC10961756; doi:10.3390/epigenomes8020012)

Table S1. Top 10 pathways for lung function and asthma acquisition (ALSPAC cohort)

FVC	FEV1	Ratio	Asthma acquisition
neurotransmitter biosynthetic process	long-chain fatty acid biosynthetic process	urea channel activity	growth hormone receptor activity
neurotransmitter metabolic process	ovarian follicle atresia	sodium:sulfate symporter activity	meiotic gene conversion
hindbrain formation	transposon integration	urea transmembrane transporter activity	growth hormone receptor complex
cerebellum formation	regulation of transposon integration	urea transmembrane transport	gene conversion
midbrain-hindbrain boundary maturation	negative regulation of transposon integration	urea transport	prolactin secreting cell differentiation
midbrain-hindbrain boundary maturation during brain development	acyl-CoA metabolic process	UTP:glucose-1-phosphate uridylyltransferase activity	taurine metabolic process
homoiothermy	thioester metabolic process	UTP-monosaccharide-1-phosphate uridylyltransferase activity	alkanesulfonate metabolic process
dopamine beta-monooxygenase activity	long-chain fatty acid metabolic process	glucose 1-phosphate metabolic process	deltoid tuberosity development
octopamine biosynthetic process	palmitic acid metabolic process	pyrimidine ribonucleotide binding	distal tubule morphogenesis
octopamine metabolic process	palmitic acid biosynthetic process	one-carbon compound transport	chiasma assembly

Figure S1. Overlap of CpGs identified in IOWBC which DNA methylation at birth are also associated with asthma acquisition (AA), and lung function measures including FEV1, FVC and FEV1/FVC ratio in ASLPAC replication cohort.

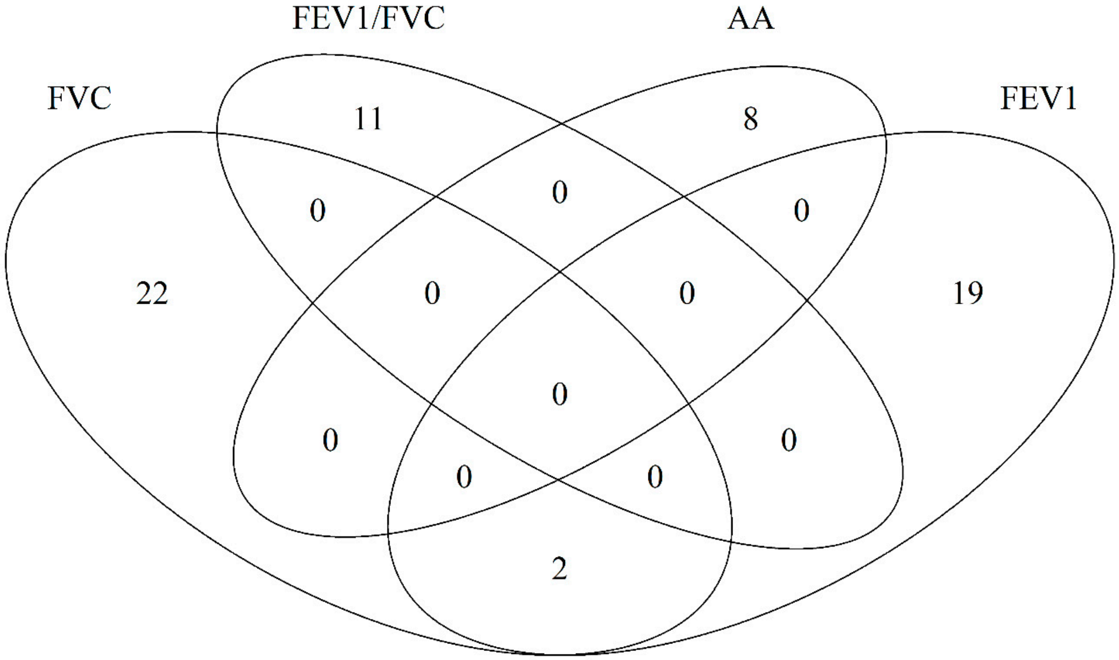

Supplement: Supplementary file 1 [file epigenomes-08-00012-s001.zip › Supplementary file S4 top 10 pathway ALSPAC.pdf]
